# Retronasal olfactory testing in early diagnosed and suspected COVID-19 patients: a 7-week follow-up study

**DOI:** 10.1007/s00405-021-06826-1

**Published:** 2021-05-13

**Authors:** Bernhard Prem, David Tianxiang Liu, Gerold Besser, Bertold Renner, Christian Albert Mueller

**Affiliations:** 1 Department of Otorhinolaryngology, Head and Neck Surgery, Vienna General Hospital, Medical University of Vienna, 1090 Vienna, Austria; 2 Institute of Experimental and Clinical Pharmacology and Toxicology, Friedrich-Alexander Universität Erlangen-Nürnberg, Erlangen, Germany; 3 Medical Faculty Carl Gustav Carus, Institute of Clinical Pharmacology, Technische Universität Dresden, Dresden, Germany

**Keywords:** Anosmia, COVID-19, Olfaction, Retronasal, Flavor

## Abstract

**Objectives:**

Olfactory dysfunction (OD) constitutes a major symptom in Coronavirus Disease 2019 (COVID-19). Yet, most data on smell loss rely on the evaluation of orthonasal olfactory performance. Therefore, we aimed to assess retronasal olfactory function (ROF) over a period of several weeks in proven and suspected COVID-19 patients.

**Methods:**

One hundred and one subjects with suspected or laboratory-proven COVID-19 participated in this study. In patients with OD no longer than 4 weeks after initial symptom onset, ROF was measured with the 7-item Candy Smell Test ten times over 7 weeks.

**Results:**

Olfactory function was decreased in the investigated patients and remained decreased over the course of 7 weeks. One-way repeated-measures ANOVA revealed no significant difference of ROF between different measurement time points. However, self-assessment of smell and flavour improved significantly (*p* = 0.013 and *p* = 0.043), but did not show complete recovery.

**Conclusion:**

The current investigation revealed significant improvements in subjective smell and flavour perception over the course of 7 weeks in proven and suspected COVID-19 patients suffering from acute OD. However, objectively measured ROF based on a screening test revealed no improvements within the same time period.

## Introduction

The Coronavirus Disease 2019 (COVID-19) pandemic remains a challenging situation since the beginning of 2020. Symptoms of infection with Severe-Acute-Respiratory-Syndrome Coronavirus-2 (SARS-CoV-2) are diverse and range from fever, cough, dyspnea, headache, fatigue, myalgia, diarrhea to olfactory dysfunction (OD) [1–4]. It is commonly acknowledged that OD can follow viral infections of the respiratory tract [5], detailed knowledge in the underlying mechanisms however is relatively sparse [6]. Beyond the overwhelming number of downsides, the current pandemic may hold the possibility of better understanding postviral OD [7].

At the beginning of the pandemic, OD was reported in 5% of COVID-19 patients [8]. More recent studies revealed that overall more than two out of three COVID-19 patients in the US or Europe suffer from OD [9, 10], with a range from 19% up to 85% [11–13].

Different studies reported higher prevalences of self-assessed OD during the COVID-19 pandemic in general [3, 14], while others revealed subjective OD in proven SARS-CoV-2 infected patients [11, 15, 16]. Until January 2021, only few studies confirmed OD by psychophysical olfactory testing. Moein et al. used the University of Pennsylvania Smell Identification Test (UPSIT) and showed that 98% of patients tested positive for COVID-19 suffer from different degrees of OD [17]. Using the identification subtest of Sniffin’ Sticks, Lechien et al. reported OD in more than 70% of SARS-CoV-2 infected patients [18]. All of these tests evaluate orthonasal olfactory function. However, retronasal olfactory testing has not been considered in COVID-19 patients, although retronasal olfaction represents a major contributor to flavour perception [19]. Furthermore, self-administered smell tests should be advocated during home isolation due to suspected or active COVID-19 infection [20].

The aim of this study was to assess retronasal olfactory function during the course of suspected or proven infection with SARS-CoV-2 no longer than four weeks after symptom onset, using validated olfactory tests, suitable for self-administration.

## Materials and methods

The present study was approved by the ethics committee of the Medical University of Vienna (EK-No.: 1339/2020) and conducted according to the guidelines of the declaration of Helsinki on biomedical research involving human subjects. Prior to participation, all subjects provided their written informed consent.

### Patients

In this study, 101 patients (72f; 29m) with a mean age of 42.0 years (standard deviation (SD): 14.0; range 18–68 years) participated. In Austria, the first SARS-CoV-2 infections were recorded on the 25th of February 2020. We consequently included patients with subjectively novel OD and diagnosed or suspected with COVID-19 infection starting from this date. Press release [21] with information in several newspapers as well as invitational notices placed at the campus helped informing potential participants. Interested subjects contacted us via E-Mail, were informed by telephone and exclusion criteria (age below 18 or above 85 years, fructose intolerance or intolerance to sorbitol (component of the retronasal smell test), dysphagia, head/neck-tumours, onset of _OD prior to 25th_ of February 2020) were checked. In addition, we excluded patients who reported head trauma, sinonasal or neurological diseases, to include only suspected (and proven) COVID-19 patients.

**Fig. 1:**
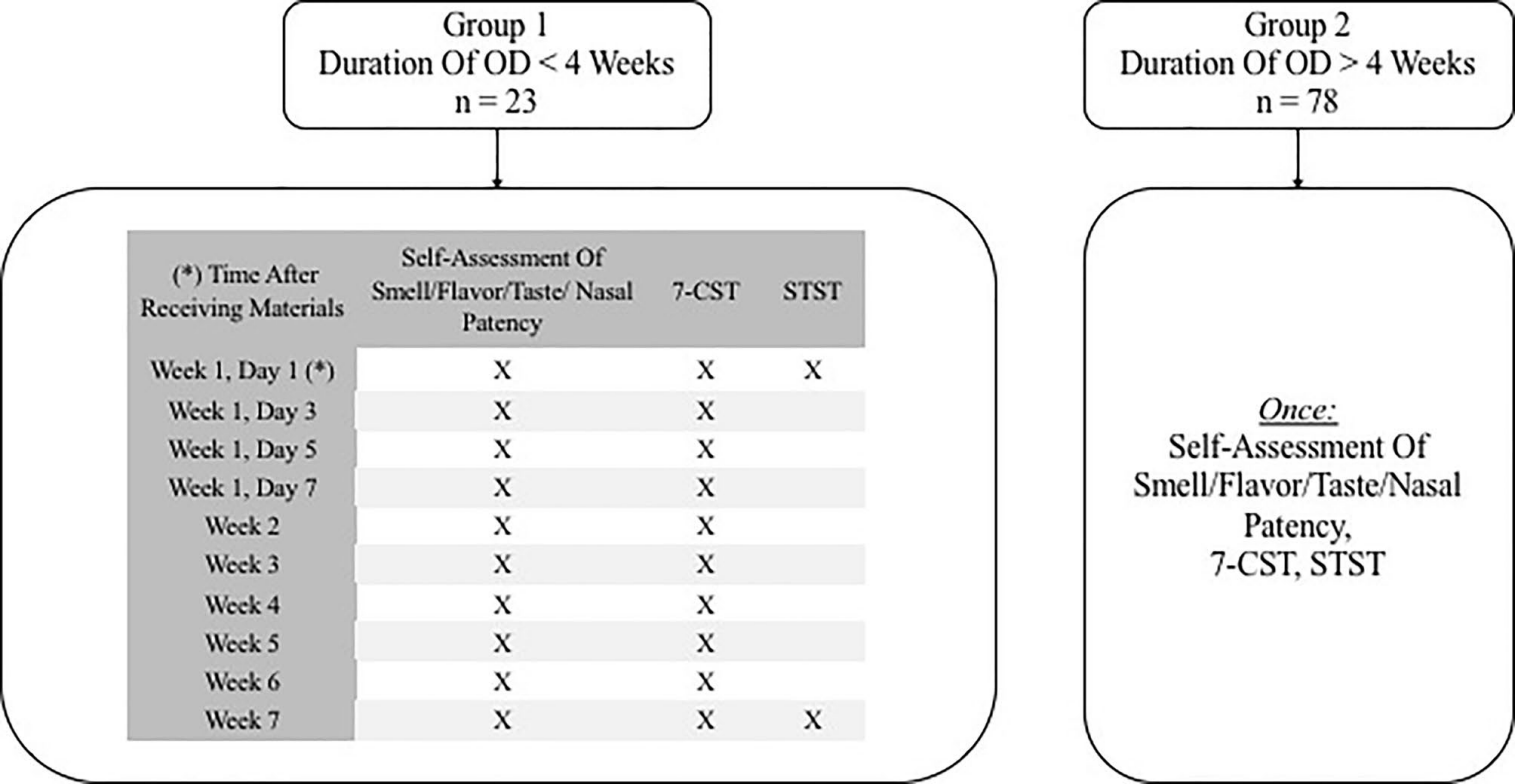
Flowchart of the procedure according to group classification. *OD* olfactory dysfunction, *7-CST* 7-item Candy Smell Test, *STST* Suprathreshold Taste Strips Test

Upon initial contact, patients were divided into two groups: Group 1 consisted of patients with onset of OD less than four weeks. Group 2 entailed patients with onset of OD longer than 4 weeks (Fig. 1). Patients of group 1 were tested repeatedly (see Fig. 1) to detect early recovery of olfactory performance [22].

### Questionnaires and tests sent by post

Subjects received a package sent by post with a detailed description of self-administration of all chemosensory tests. Additional detailed instructions about the conduct of chemosensory tests were provided by telephone.

The package of group 1 contained general questions [age, gender, clinical symptoms), two questionnaires (Importance of Smell (IOS) and Questionnaire of Olfactory Disorders (QOD)] [23, 24], ten sets of a retronasal screening smell test (the 7-item-Candy-Smell-Test (7-CST), [25, 26]) and two sets of a Suprathreshold Taste Strips Test (STST) [27]. Patients were instructed to perform the 7-CST four times within the first week and once a week for another 6 weeks to record early regeneration of smell function [22]. STST was performed twice (together with the first and the last 7-CST).

Group 2 received a package containing the same general questions and questionnaires as well as one 7-CST and one STST.

After performing all chemosensory tests and questionnaires, completed materials were returned by mail.

### Self-assessment of smell, flavour and taste function, and nasal patency

Investigated subjects were asked to rate their self-perceived chemosensory function of smell (SAS), flavour (SAF) and taste (SAT) on a scale from 1 (very bad) to 10 (very good) prior to every olfactory testing procedure. Upon inclusion, participants were informed about the chemosensory functions to distinguish smell, flavour and taste.

Furthermore, patients were also asked to rate SAS, SAF and SAT prior to COVID-19 infection and symptom onset based on the above-mentioned scale. Even though previous studies provided evidence that reduced olfactory function is not associated with obstruction of the nose in COVID-19 patients [11, 28–30], we also assessed self-perceived nasal patency (SANP) prior to each psychophysical chemosensory testing.

### Chemosensory testing

To evaluate retronasal olfactory function, we used the screening test (7-CST) of the Candy Smell Test (CST) [25]. Flavoured candies are placed separately on the tongue and have to be identified out of four different answers according to a forced-choice principle. In between the candies, the participants rinse the mouth with water or take a sip of water. The exact procedure is described elsewhere. [25] The CST is suitable for self-administration and postal distribution [26]. For the 7-CST seven candies have to be identified among 7 answers in a non-forced-choice procedure (thus, including the possibilities to choose “no flavour” or “undefinable”). Hence, the maximum obtainable score is 7. In this setting 0 or 1 points on the 7-CST most likely represent anosmia, and scores of 5 or higher most likely represent normosmia, whereas scores between 2 and 4 most likely denote hyposmia [26].

The gustatory function was assessed in a screening fashion using the STST with the four highest concentrations of each taste (suprathreshold testing) [27]. Small paper strips soaked in different concentrations of taste solutions (e.g., sodium chloride, or saccharose) are placed on the tongue. After closing the mouth the tested subjects has to select one out of five answers (sweet, sour, salty, bitter, no taste). After each taste strip, the participants rinses the mouth with tap water. Besides four taste strips of different concentrations of each quality (sweet, sour, bitter, salty), two blanks complete a full set of 18 taste strips. Detailed procedural explanations are written elsewhere [27] and also self-administration has been advocated [31].

### Statistical analysis

Statistical analysis and visualization of data were performed using IBM SPSS 26.0 (IBM Corp., Armonk, NY, USA) and Graph-Prism 8.4.3 (GraphPad Software, Inc., La Jolla, CA, USA). Normality of data distributions was analyzed based on histograms. One-way repeated-measures analysis of variance (ANOVA) was used for multiple group comparisons, followed by Tukey’s post hoc tests. Pearson’s correlation coefficient (*r*) was used for bivariate correlations. A *p* value < 0.05 was considered for statistical significance.

## Results

Nineteen patients (82.6%) of group 1 (patients with OD no longer than four weeks) were tested positive for SARS-CoV-2 based either on polymerase chain reaction (PCR) or antibodies (AB) against SARS-CoV-2, four participants (17.4%) were not tested initially during the acute phase of the disease. In 2 out of these 4 patients, no AB were detected either 163 or 186 days after the onset of OD. On average, participants from group 1 tested themselves 24 days (SD: 10.6) after the onset of OD. Besides OD, myalgia (56.5%), cough (52.2%) and fever (43.5%) have been the most common symptoms within this group (Table 1).

**Table 1:** Descriptive statistics of Group 1

Descriptive statistics	
*N*	23
Gender	Female: 18 Male: 5
Age (in years)	Mean: 41.2 (SD: 11.7; range: 24–57)
Duration of OD until first testing (in days)	Mean: 23.9 (SD: 10.6; range: 5–44)
Tested^a^ for COVID-19	Not tested: 2
	Tested positive: 19
	Tested negative (AB): 2
Smoker	Never-smoker: 11
	Former smoker: 11
	Smoker: 1
BMI (in kg/m^2^)	Mean: 25.0 (SD: 3.4; range 19.5–34.0)
Other symptoms	
Cough	12 (52.2%)
Sore throat	9 (39.1%)
Fever	10 (43.5%)
Dyspnea	5 (21.7%)
Rhinitis	6 (26.1%)
Myalgia	13 (56.5%)
Self-assessment before OD	
Smell	Mean: 9.6 (SD: 0.6; range 8–10)
Flavour	Mean: 9.4 (SD: 0.8; range 8–10)
Taste	Mean: 9.6 (SD: 0.7; range 8–10)
Nasal patency	Mean: 9.0 (SD: 1.2; range 6–10)

*SD* standard deviation, *BMI* body mass index (kg/m^2^), *OD* olfactory dysfunction

^a^Using either polymerase-chained reaction (PCR) or antibodies (AB) against SARS-CoV-2

In group 2 (patients with OD longer than 4 weeks), fifty-two subjects (66.7%) were tested positive for SARS-CoV-2—either PCR or AB—and twenty-six participants (33.3%) have not been tested for SARS-CoV-2 during the acute phase of disease. In 6 out of these 26 participants, no AB against SARS-CoV-2 were detected, on average, 186 days (range 157–221 days) after the onset of OD. The mean duration between the onset of OD and testing day of group 2 was 47 days (SD: 31.0). Fever (42.3%), cough (41.0%), rhinitis (39.7%) and myalgia (39.7%) account for the most common symptoms besides OD (Table 2). To depict differences in the self-assessed and psychophysical chemosensory function of patients from group 1, we performed one-way repeated-measures ANOVA (rm-ANOVA) with appropriate post hoc tests whenever applicable.

**Table 2:** Descriptive statistics of Group 2

Descriptive statistics	
*N*	78
Gender	Female: 54 Male: 24
Age (in years)	Mean: 42.2 (SD: 14.7; range 18–68)
Tested^a^ for COVID-19	Not tested: 20 Tested positive: 52 Tested negative (AB): 6
Smoker	Never-smoker: 45
	Former smoker: 24
	Smoker: 9
BMI (in kg/m^2^)	Mean: 24.3 (SD: 4.3; range 17.6–45.8)
Other symptoms	
Cough	32 (41.0%)
Sore throat	27 (34.6%)
Fever	33 (42.3%)
Dyspnea	19 (24.4%)
Rhinitis	31 (39.7%)
Myalgia	31 (39.7%)
Self-assessment before OD	
Smell	Mean: 9.4 (SD: 1.2; range 8–10)
Flavour	Mean: 9.3 (SD: 1.2; range 8–10)
Taste	Mean: 9.4 (SD: 1.3; range 8–10)
Nasal patency	Mean: 9.0 (SD: 1.6; range 6–10)
Chemosensory tests	
7-CST (in points)	Mean: 3.2 (SD: 2.0; range 0–7)
STST (in points)	Mean: 3.7 (SD: 0.7; range 1–4)

*SD* standard deviation, *BMI* body mass index (kg/m^2^), *OD* olfactory dysfunction

^a^Using either polymerase chain reaction (PCR) or antibodies (AB) against SARS-CoV-2

**Fig. 2:**
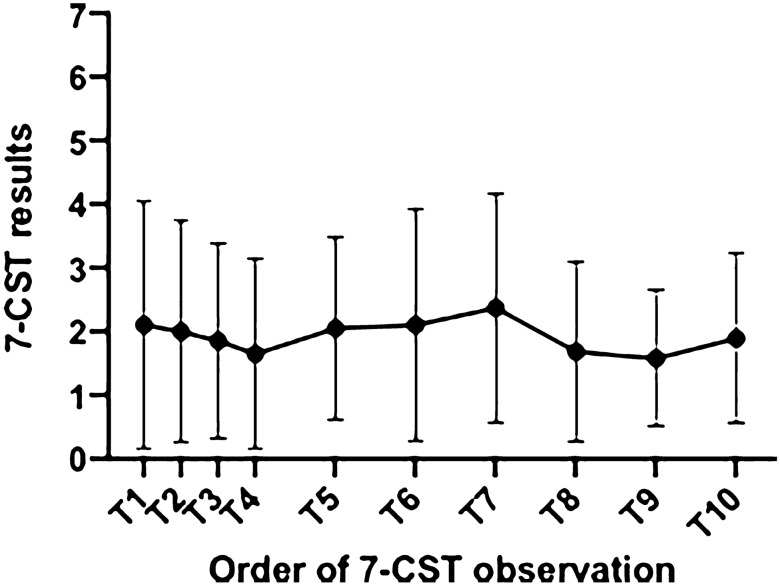
Mean results (± standard deviation) of 7-CST in course of 10 tests (7 weeks), Group 1 (*n* = 23). Tests 1–4: first week. Test 5: end of second week. Test 6: end of third week, etc. Test 10: end of seventh week. *7-CST* 7-item Candy Smell Test

One-way rm-ANOVA for 7-CST results in course of 7 weeks revealed no significant differences [*F* (4.64, 85.51) = 0.96; *p* = 0.44] (Fig. 2). Furthermore, the comparison of long-term results (at the beginning and the end of the 7 weeks observation period) of STST was not significantly different (*p* = 0.07).

**Fig. 3:**
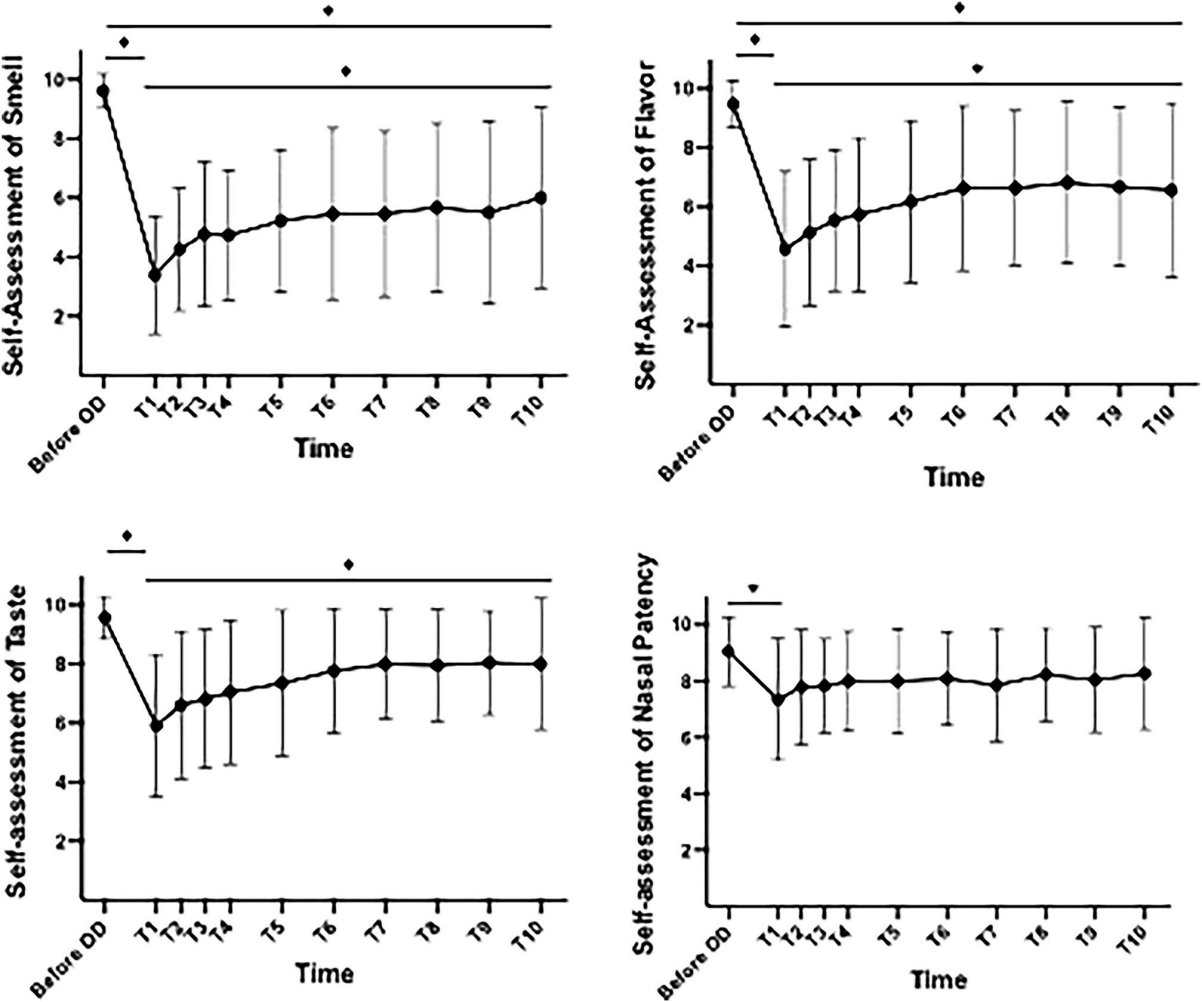
Means (± standard deviation) of self-perceived smell, flavour, and taste function and nasal patency on a scale from 1 (very bad) to 10 (very good)—before OD and during the course of psychophysical olfactory testing of group 1. *Significant difference (if applicable, marked only between before OD and T1, before OD and T10, or T1 and T10). *OD* olfactory dysfunction, *T1-T10* self-administered psychophysical olfactory testing 1–10

One-way rm-ANOVA of SAS revealed significant differences across the observational period [*F* (3.00, 63.97) = 26.59; *p* < 0.002] (Fig. 3). Tukey’s post hoc test revealed significant improvement (*p* = 0.01) of SAS from the first (Test 1) to the last investigation (Test 10). Similarly, one-way rm-ANOVA of SAF also revealed significant differences [*F* (3.59, 76.46) = 18.15; *p* < 0.02] (Fig. 3). Tukey’s post hoc test showed significant improvement of SAF between the first and the last test (*p* = 0.04). One-way rm-ANOVA of SAT in course of seven weeks revealed significant differences [*F* (4.15, 88.41) = 13.08; *p* < 0.001]. Tukey’s post hoc test showed significant deterioration of SAT after the onset of OD. Nevertheless, subjective reconvalescence was only observed for taste function (Fig. 3).

In order to detect differences between proven (*n* = 71) and highly suspected (*n* = 30) SARS-CoV-2 infections, we compared subjective and objectively evaluated chemosensory function. Applying unpaired Student’s *t* test or Kolmogorov–Smirnov test between self-assessed (SAS, SAF, and SAT) and psychophysically tested (7-CST and STST) function of chemosensory function between these proven and suspected COVID-19 patients revealed no significant difference (*p* > 0.05, respectively).

To depict the relationship between self-assessed and psychophysical tested olfactory performance, we applied Pearson’s correlation coefficient in group 1. Significant correlations between 7-CST and SAS were seen at test 6 (*r*_23_ = 0.60; *p* = 0.007), test 7 (*r*_23_ = 0.68; *p* = 0.001), test 9 (*r*_23_ = 0.51; *p* = 0.027) and test 10 (*r*_23_ = 0.63; *p* = 0.002) of group 1 (Table 3). Significant correlation between 7-CST and SAF (*r*_23_ = 0.52; *p* = 0.017) was observed only for the last test (Table 3). In group 2 we found significant correlations between 7-CST and SAS (*r*_78_ = 0.61; *p* < 0.001), and between 7-CST and SAF (*r*_78_ = 0.43; *p* < 0.001). Furthermore, Pearson’s correlation of the whole sample revealed a significant correlation between SAF and SAT (*r*_101_ = 0.73; *p* < 0.001), as well as between SAS and SAT (r_101_ = 0.48; p < 0.001), and between SAS and SAF (*r*_101_ = 0.65; *p* < 0.001).

**Table 3:** Pearson’s correlation (*r*_23_) between 7-CST results and either SAS or SAF during the course of psychophysical olfactory testing

Correlation between 7-CST and either SAS or SAF	Test 1	Test 2	Test 3	Test 4	Test 5	Test 6	Test 7	Test 8	Test 9	Test 10
Group 1										
SAS	0.35	0.18	0.05	0.27	0.15	0.60*	0.68*	0.36	0.51*	0.63*
SAF	− 0.15	− 0.18	− 0.14	0.10	0.27	0.38	0.30	0.05	0.17	0.52*

*7-CST* 7-item Candy Smell Test, *SAS* self-assessment of smell, *SAF* self-assessment of flavour

*Significant correlation (*p* < 0.05)

Results of STST and SAT at the beginning (*r*_23_ = 0.49; *p* = 0.018) and the end (*r*_23_ = 0.58; *p* = 0.006) of the 7 weeks period correlated significantly in group 1. Group 2 showed only weak correlation between STST results and SAT (*r*_78_ = 0.24; *p* = 0.035).

The mean score of SANP before acute chemosensory dysfunction was 9.0 (SD = 1.3) in group 1. One-way rm-ANOVA [*F* (4.69, 100.8) = 3.70; *p* = 0.005] showed significant difference across observational period. Tukey’s post hoc test revealed a significant difference only between SANP before acute onset of OD and SANP during the first test (Fig. 3). However, Pearson’s correlation revealed no significant correlation between SANP and either SAS, SAF or 7-CST at any time.

Comparing SANP before OD and during test procedure in group 2 showed a significant difference (*p* < 0.001) after the onset of OD. A weak significant correlation between SANP and SAS was observed (*r*_78_ = 0.27; *p* = 0.018); however, no significant correspondence between SANP and either SAF or 7-CST was found.

## Discussion

In contrast to current publications evaluating orthonasal olfactory performance in COVID-19 patients, data regarding retronasal olfactory function and its course during acute phase of the disease are missing.

We could find the following main results: first, decreased olfactory function was found in the investigated patients measured by a validated retronasal smell test. Second, no significant differences in retronasal olfactory performance could be observed in course of seven weeks using a retronasal smell test made of candies. Third, decreased subjective smell and flavour perception increased during the observational period but did not reach normal levels. Fourth, in addition to reduced smell function, we observed a significant decrease in subjective gustatory function. And fifth, no considerable correlations between self-assessed nasal patency and either retronasal smell test results or subjective function of smell or flavour were found.

A few other studies evaluated follow-up assessment of OD by utilizing validated psychophysical olfactory tests [32–36]. Eighty-two patients suffering from OD due to COVID-19 have been re-tested one or four weeks after the onset of OD using the University of Pennsylvania Smell Identification Test (UPSIT) [35]. Approximately 63% were normosmic again. Nevertheless, in comparison to normative data, those patients still revealed worse olfactory function compared to the age- and gender-related mean of healthy population.

Another longitudinal study with a 2-month follow-up of patients suffering from OD, caused by SARS-CoV-2, revealed that 75 to 85% regained normal olfactory function applying SSI [36]. Five weeks after the onset of OD Le Bon et al. observed that 37% still suffer from OD by applying the complete “Sniffin’ sticks” set [34]. Forty-six percent of all participants showed two months after the onset of OD pathological orthonasal olfactory function using the TDI [33]. However, due to the fact that flavour perception during food intake plays a major role in the individual quality of life, precise observation of retronasal olfactory function, especially during the COVID-19 pandemic, is crucial [37].

In the present study, no significant improvement of retronasal olfactory function within seven weeks was observed. With these results, we want to consider—in contrast to many other publications stating that OD lasts for only 4 weeks in most cases—that OD may last longer in more subjects than initially assumed. Nevertheless, patients reported subjective improvement of smell and flavour function. Due to this discrepancy, affected patients must be warned against hazardous situations—e.g. spoiled foodstuffs—and olfactory training should be considered in a medical consultation.

Cross-sectional observation (group 2) of retronasal olfactory performance showed 7-CST results out of the normal range in 58 out of 78 patients (74%). This compares to other cross-sectional studies evaluating olfactory performance by orthonasal smell tests. The prevalences of OD in COVID-19 patients obtained by Sniffin’ Sticks ranged from 37% five weeks after onset of OD [34], to 46% after two months [33] and up to 71–83% within the first month of OD [18]. Applying the UPSIT in Iran showed that even 98% of COVID-19 patients suffered from OD [17]. Using the CCCRC, Vaira et al. revealed a prevalence of OD in 83% of SARS-CoV-2 patients [38].

In the present investigation, a screening test of retronasal olfactory function was advocated. Two other studies proved the applicability of short screening tests to detect reduced olfactory function in SARS-CoV-2-infected patients. Seventy-two percent of all self-reported OD in COVID-19 patients were proven by applying the 4-item pocket test [39]. Lima et al. revealed a significant difference in olfactory performance between COVID-19 patients with self-reported OD and a control group by utilizing the Quick Smell Identification Test [40]. The results of these two studies assume the applicability of short, psychophysical olfactory screening tests to evaluate olfactory performance. However, so far no other studies have evaluated retronasal olfactory function in COVID-19 patients.

Significant decrease in self-assessed smell and flavour perception in group 1 were reported after the onset of symptoms. During seven weeks of observation, self-assessment improved significantly. However, we could not objectify this subjective improvement with the 7-CST. One explanation may be that the 7-CST is a screening test and thus, slight changes in olfactory function remained undetected. Another reason may be the poor correlation between subjective and tested olfactory function in general [41, 42] and particular in COVID-19 patients [33]. However, significant differences between subjective function of smell and flavour before the onset of symptoms, on the first day of testing and after 7 weeks were observed. Thus, self-perceived function of smell and flavour improved significantly in course of disease, but still seemed to be significantly worse in comparison to self-assessment before the onset of symptoms, which we could confirm by the results of decreased smell tests.

Similar to Le Bon et al. [34], we observed acute decrease of subjective gustatory function during the onset of symptoms. Nevertheless, after 7 weeks of observation, there was no significant difference in taste function compared to the status before the onset of disease, revealing subjective reconvalescence of taste function. Repeated measures of tested taste function could not confirm this finding during the course of disease. However, due to the relatively short testing procedure (screening method), the test might not be able to detect subtle changes in taste performance. In summary, observing decreased self-assessed function of smell, flavour and taste all together in patients, support the suggestion of the close connection of the human chemical senses [43], or lead to the assumption that SARS-CoV-2 affects all chemosensory organs.

Due to the fact that not all patients provided proven infection with SARS-CoV-2 and, therefore, suspected COVID-19 patients might bias data, we compared those two groups. Neither self-assessed nor psychophysically evaluated chemosensory function showed significant differences between proven and suspected COVID-19 patients. Thus, complete data of the present study seem to be valid and comparable without obvious bias of the results. However, it seems to be feasible that sudden onset of smell loss in those patients also occurred due to infection with SARS-CoV-2.

During the course of observation within 7 weeks, we found no considerable correlation between subjective nasal patency and either SAS, SAF or 7-CST results of group 1. Thus, OD in SARS-CoV-2 might not be caused by impaired nasal patency. According to current studies, our results seem to be representative [11, 28–30].

## Conclusion

The present investigation showed decreased olfactory function with no significant improvement of retronasal olfactory function using 7-CST in proven and suspected COVID-19 patients. However, subjective smell and flavour perception improved during the course of 7 weeks, but still differed significantly from before the onset of OD. In addition to current publications, we found no considerable correlation between subjective nasal patency and either (retronasal) olfactory function, or subjective assessment of smell or flavour perception in this study.
